# Passing Events and Topics

**Published:** 1920-10-09

**Authors:** 


### Passing Events and Topics.

### Founder's Day at Putney.

Founder's Day will be celebrated at the Royal Hospital and Home for Incurables, Putney, on Tuesday, November 30.

### The Great Northern Central Hospital.

The Flag Day held at Islington on September 15 in aid of the Great Northern Central Hospital Extension Fund realised over £270, which was a satisfactory result in view of the wet weather. To the same Fund the Mayor of Islington has received a donation of fifty guineas from the Arsenal Football Club.

### Repayment of Spirit Duty.

The Ministry of Health and the Scottish Board of Health invite applications from voluntary hospitals for grants in respect of payment of spirit duty for medical and surgical purposes during 1919.

